# A knock-in translational reporter for NLP-29 reveals AMP secretion to the apical extracellular matrices following epidermal damage in *Caenorhabditis elegans*

**DOI:** 10.17912/micropub.biology.001435

**Published:** 2025-03-29

**Authors:** Nathalie Pujol, Henrik Bringmann

**Affiliations:** 1 Centre d’Immunologie de Marseille-Luminy, Aix-Marseille Université, Marseille, Provence-Alpes-Côte d'Azur, France; 2 Turing Centre for Living Systems, Centre National de la Recherche Scientifique, Marseille, France; 3 Center for Molecular and Cellular Bioengineering (CMCB), Biotechnology Center (BIOTEC), TU Dresden, Dresden, Saxony, Germany

## Abstract

Antimicrobial peptides (AMPs) are small proteins produced and secreted as part of the innate immune response to infection and wounding. They target pathogens and can also function as signalling molecules, for example, promoting sleep in response to injury in *C. elegans* . A transcriptional reporter transgene for *nlp-29* has been pivotal in studying AMP gene expression and regulation, but to understand AMPs antimicrobial and signalling roles, protein expression and trafficking needs to be monitored. We have now created a knock-in translational reporter allele for *nlp-29* , with NLP-29 fused to mKate2, that enables visualisation of this secreted AMP. Using the NLP-29::mKate2 reporter, we demonstrate that NLP-29 is secreted into the cuticle upon genetic or physical cuticle damage. NLP-29::mKate2 will therefore be a valuable tool for visualising the secretion of this peptide in *C. elegans* and thus to dissect the different roles of this key AMP.

**Figure 1. Visualization of NLP-29 secretion using an mKate2 fusion knock-in nlp-29 allele:**
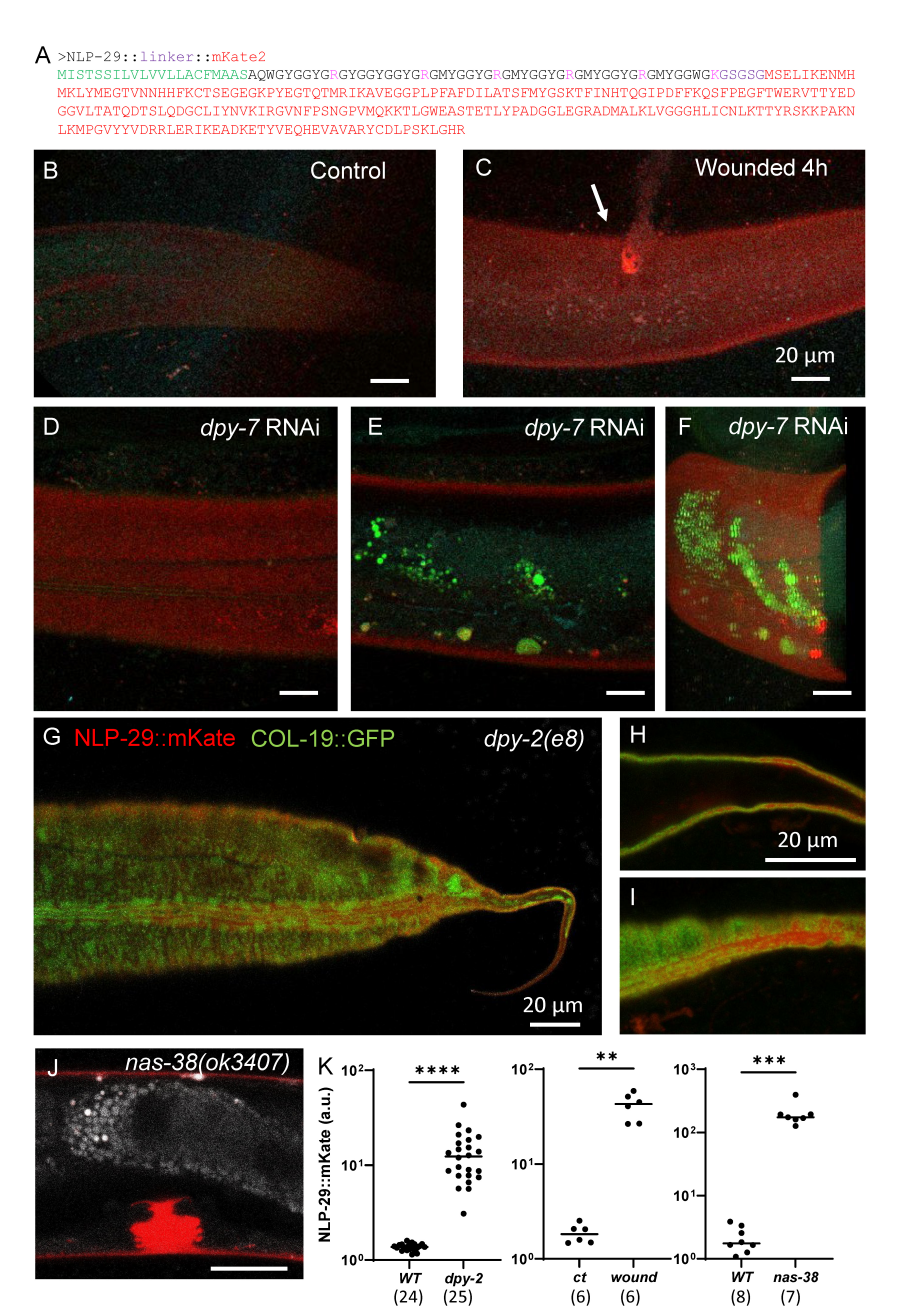
A) Predicted protein sequence of the NLP-29::linker::mKate2 (hereby NLP-29::mKate2) translational fusion protein. The signal sequence is shown in green. The peptide sequence is highly repetitive yet does not contain any dibasic sites and is hence predicted to not be further processed, allowing for AMP visualisation using the fluorescent mKate2 tag shown in red. B-C) Representative confocal images of the NLP-29::mKate2 reporter in a young adult worm in the wild type in control condition (B) and 4 hours after needle wounding (C); n > 10; scale bar 20 µm. D-F) Representative confocal images of the NLP-29::mKate2 reporter in a young adult worm upon RNAi inactivation of the *dpy-7* furrow collagen gene. Two different planes are shown: one at the level of the cuticle (D) and another at the level of the intestine (E). Additionally, a 3D rendering (F) provides a comprehensive view of these anatomical structures; autofluorescence is visualised in green; n > 10; scale bar 20 µm. G-I) Representative confocal images of the NLP-29::mKate2 and COL-19 ::GFP reporters in a young adult *dpy-2 ( e8 )* worm. Three different imaging planes of the same animal are presented to visualise the co-localisation of NLP-29::mKate2 and COL-19 ::GFP in the cuticle; n > 10, scale bar 20 µm. J) A representative confocal image of the NLP-29::mKate2 reporter in a *nas-38 ( ok3407 )* L4.4 worm allows visualisation of the localisation of NLP-29::mKate2 in both the vulva lumen and the cuticle; n > 10; scale bar 20 µm. K) Quantification of the red NLP-29::mKate2 fluorescence in different mutant backgrounds or conditions in young adult worms. Statistical comparisons were made using the unpaired Mann Whitney U test by comparing the control (ct = unwounded control, wt = wild type) with the experimental conditions ( *dpy-2* mutation, wounding, or *nas-38* gain-of-function mutation); ** *p* < 0.01; *** *p* < 0.001; **** *p* < 0.0001.

## Description

The skin serves as the outermost defence against pathogens. Across species, damage or infection of the skin triggers an innate immune response in the underlying epidermis, involving a cascade of signalling molecules, leading to the production of antimicrobial peptides (AMPs) (Hanson and Lemaitre, 2020; Lai and Gallo, 2009; Martineau et al., 2021). These AMPs act against pathogens by disrupting membrane structures (Lai and Gallo, 2009), and also play roles in signalling, such as promoting sleep in response to injury (Sinner et al., 2020; Toda et al., 2019) and neuron degeneration (E et al., 2018).

*C. elegans* is an important model for AMP research due to its lack of an adaptive immune system, its amenability to genetic manipulation, and its ability to be wounded or infected by various pathogens (Couillault et al., 2004; Martineau et al., 2021; Pujol et al., 2008a; Pujol et al., 2008b; Taffoni et al., 2020). Innate immune responses of the epidermis involve the production of two main classes of AMPs: 1) neuropeptide-like peptides (NLP) AMPs, regulated by PMK-1 p38 MAP kinase signalling (Pujol et al., 2008a), and 2) caenacin (CNC) AMPs, regulated by the TGF-β homolog DBL-1 (Martineau et al., 2021; Zugasti and Ewbank, 2009). The mature peptides are basic and rich in glycine and aromatic amino acids and harbour a conserved motif QWGYG just C-terminal to the predicted signal sequence cleavage site (Couillault et al., 2004). Transcriptional reporters based on multi-copy arrays have been instrumental for visualising AMP expression and identification of the signalling pathways leading to their regulation, like *frIs7 (nlp-29p::GFP)* (Pujol et al., 2008a), and *cnc-2 p::GFP* (Zugasti and Ewbank, 2009), as reviewed in (Martineau et al., 2021). While very few antimicrobial proteins have been directly tagged, including LYS-1 (Mallo et al.,2002), SPP-3 and SPP-12 (Hoeckendorf el al., 2012a&b) *,* and ASP-3 and ASP-4 (Wong et al., 2007), AMPs have not yet been visualised in *C. elegans* , and it is not known, for example, whether they are secreted apically or in a basolateral manner.

To investigate AMP secretion, we designed and created knock-in translational fusion alleles of *cnc-2* and *nlp-29* with *mKate2* . We used a linker sequence (GSGSG) to attach mKate2 to the C-terminus of either AMP gene. We codon-optimized the linker and mKate2 sequence for optimal expression and inserted two synthetic introns into the mKate2 coding sequence (Redemann et al., 2011) ( Figure 1A ; methods). This optimised construct was then integrated into the endogenous *cnc-2* and *nlp-29* loci using CRISPR-Cas9 to create *cnc-2 ::mKate2* and *nlp-29 ::mKate2* , respectively. The CNC-2 and NLP-29 preproteins contain a cleavable N-terminal signal sequence but lack the dibasic residues that act as sites of internal proteolytic cleavage (Couillault et al., 2004; Nathoo et al., 2001). This suggests that the mature peptides are not further processed and that the AMP would remain connected to the mKate2 sensor after secretion, which should allow for visualising the AMPs *in vivo* .

We imaged mKate2 fluorescence in adult worms under baseline conditions and after cuticle damage, which triggers innate immune responses involving the upregulation of *cnc-2* and *nlp-29* expression (Dodd et al., 2018; Meng et al., 2020; Pujol et al., 2008a). For damaging the cuticle, we used either a glass needle (Pujol et al., 2008a; Xu and Chisholm, 2011) or the inactivation of furrow collagens (Aggad et al., 2023; Dodd et al., 2018; Pujol et al., 2008b; Sundaram and Pujol, 2024; Taffoni et al., 2020). We did not detect fluorescence from *cnc-2 ::mKate2* under these conditions, potentially because the endogenous level of expression is too low, which is in line with the results obtained with the transcriptional reporter (Zugasti and Ewbank, 2009). *nlp-29 ::mKate2* also showed no detectable fluorescence under baseline conditions. This is consistent with low level of expression observed in several transcriptomic studies (Couillault et al., 2004; Dodd et al., 2018; Hendriks et al., 2014). A clear signal in the cuticle was, however, observed after epidermal wounding in the wild type ( Figure 1 C) and upon inactivation of the furrow collagen *dpy-7* using RNAi ( Figure 1 E-K). We confirmed that the NLP-29::mKate2 signal localized to the cuticle using a cuticle collagen marker, COL-19 ::GFP. In the furrow collagen *dpy-2 ( e8 )* mutant, both signals co-localised ( Figure 1 I-K). We confirmed this localisation in a gain-of-function mutant of *nas-38* ( *nas-38 ( ok3407 ),* Figure 1J ), which represents another condition in which *nlp-29* is known to be up-regulated (Sinner et al., 2020). Interestingly, NLP-29 is also found to be secreted in the vulva lumen during the L4 stage in *nas-38* mutants ( Figure 1J ) or furrow-less mutants. The quantification of the reporter signal in the cuticle of all three conditions — furrow-less *dpy-2* mutants, gain-of-function *nas-38* mutants, and wounded wild-type worms — shows a significant increase in the NLP-29::mKate2 fluorescence signal ( Figure 1K ). The localisation of NLP-29::mKate2 in the apical extracellular matrices aligns with its antimicrobial function.

Given the known signalling function of AMPs such as NLP-29 , one would predict that some of the protein should be released basolaterally into the pseudocoelom to then diffuse and act on the nervous system (E et al., 2018; Sinner et al., 2020). We did not, however, observe NLP-29::mKate2 in the pseudocoelom or coelomocytes. This lack of detection of NLP-29::mKate2 internally could reflect a low level basolateral secretion, and/or its dilution or degradation in the pseudocoelom. It should also be noted that any detection in coelomocytes is complicated by their intrinsic red autofluorescence. We cannot, therefore, rule out potential basolateral secretion that might be important for NLP-29 's signalling to neurons (E et al., 2018; Sinner et al., 2020). Our observations suggest that NLP-29 's antimicrobial functions that rely on direct action on pathogen membranes could require higher peptide concentrations compared to signalling functions, which are amplified via G protein-coupled receptors (Sinner et al., 2020; Zugasti et al., 2014). Hence, more sensitive detection methods with brighter fluorophores, or immunohistochemistry, might be needed to visualise the putative internal secretion of NLP-29 or other AMPs in the future.

This NLP-29::mKate2 fusion protein is, to our knowledge, the first reporter allowing an AMP's localisation to be monitored in *C. elegans* and will be a valuable tool for future studies.

## Methods

**Nematode strains**

All *C. elegans* strains were maintained on nematode growth medium (NGM) and fed with *E. coli* OP50 , as described (Stiernagle, 2006). The TP12 *kaIs12 [ COL-19 ::GFP]* (Thein et al., 2003) and *dpy-2 ( e8 )* (Brenner, 1974) strains were obtained from the CGC. The IG2117 *nlp-29 ( syb1965 )[NLP-29::linker::mKate2]) V; kaIs12 [ COL-19 ::GFP],* IG2115 *dpy-2 ( e8 ) II; nlp-29 ( syb1965 )[NLP-29::linker::mKate2]) V; kaIs12 [ COL-19 ::GFP]* and IG2000 *nas-38 ( ok3407 ) X; nlp-29 ( syb1965 )[NLP-29::linker::mKate2]) V* were obtained by conventional crosses.

**Generation of reporter knock-in alleles for *cnc-2* and *nlp-29***

We designed the *linker* and *mKate2* sequence in silico, and the corresponding DNA was synthesized by a commercial provider. Using this synthetic DNA as a template, the endogenous loci of *cnc-2* and *nlp-29* were edited using CRISPR-Cas9 by a commercial service provider (Sunybiotech) to introduce the gene edits. Synonymous mutations were also introduced during the gene editing process to prevent recutting by the nuclease (synonymous changes for *nlp-29* are labelled in bold in the sequence below). The allele for *cnc-2* was constructed using the identical *linker::mKate2* sequence, with the sequence GGAATGCT **C** ATGGGCAAG mutated to GGAATGCT **G** ATGGGCAAG (a silent mutation) to remove the PAM site. The editing resulted in two strains, both of which were superficially normal and homozygous viable.

PHX1965 *nlp-29 ( syb1965 [::linker::mKate2])* with the following sequence (the linker is underlined, the synonymous mutation is labelled in **bold** )

ATGATTTCAACCTCTTCAATTCTTGTTCTTGTCGTCCTTCTCGCCTGCTTCATGGCTGCCAGCGCACAATGGGGATATGGAGGATATGGAAgtgagtttttttgtgctttttgacttatctcaaaaaaagtagatcattcacacatattttcattttcagGAGGATATGGAGGATATGGTGGATACGGACGAGGAATGTATGGAGGCTATGGACGCGGAATGTATGGTGGATATGGACGTGGAATGTACGGAGGATACGGACGCGGAATGTATGGAGG **T** TGGGGAAAG GGATCCGGATCCGGA ATGTCCGAGCTCATCAAGGAGAACATGCACATGAAGCTCTACATGGAGGGAACCGTCAACAACCACCACTTCAAGTGCACCTCCGAGGGAGAGGGAAAGCCATACGAGGGAACCCAAACCATGCGTATCAAGgtaagtttaaacatatatatactaactaaccctgattatttaaattttcagGCCGTCGAGGGAGGACCACTCCCATTCGCCTTCGACATCCTCGCCACCTCCTTCATGTACGGATCCAAGACCTTCATCAACCACACCCAAGGAATCCCAGACTTCTTCAAGCAATCCTTCCCAGAGGGATTCACCTGGGAGCGTGTCACCACCTACGAGGACGGAGGAGTCCTCACCGCCACCCAAGACACCTCCCTCCAAGACGGATGCCTCATCTACAACGTCAAGATCCGTGGAGTCAACTTCCCATCCAACGGACCAGTCATGCAAAAGAAGACCCTCGGATGGGAGGCCTCCACCGAGACCCTCTACCCAGCCGACGGAGGACTCGAGGGACGTGCCGACATGGCCCTCAAGCTCGTCGGAGGAGGACACCTCATCTGCAACCTCAAGgtaagtttaaacatgattttactaactaactaatctgatttaaattttcagACCACCTACCGTTCCAAGAAGCCAGCCAAGAACCTCAAGATGCCAGGAGTCTACTACGTCGACCGTCGTCTCGAGCGTATCAAGGAGGCCGACAAGGAGACCTACGTCGAGCAACACGAGGTCGCCGTCGCCCGTTACTGCGACCTCCCATCCAAGCTCGGACACCGTTAAatacatggataaccatctattaataatttgaaaatctcatttcgttatgtaacaatgcgttgtacatatcctgatttctcacttttttcttgaataaaaacttgcataat

PHX1939 *cnc-2 ( syb1939 [::linker::mKate2])* .

The alleles were Sanger sequenced, confirming that the intended edits had been successfully introduced.

The strain PHX1965 is available at the *Caenorhabditis* Genetics Center (CGC). PHX1939 is available upon request.

**Confocal microscopy**

Worms were mounted on a 2 % agarose pad, in a drop of 1 mM levamisole in 50 mM NaCl. Images were acquired during the following 60 min, using a Zeiss LSM880 confocal laser scanning microscope with a Plan-Apochromat Oil DIC M27 40×/1.4 objective and the acquisition software Zen. Pinhole size was set to 1 AU. Samples were illuminated with 488 nm (GFP) and 561 nm (mKate2) with constant laser power, with 4 lines accumulation and 750 gain settings. Spectral imaging combined with linear unmixing was used to separate the autofluorescence (Aggad et al., 2023). Quantification of the NLP-29::mKate2 red signal was performed using Fiji. The worm cuticle was automatically segmented based on the COL-19 ::GFP green signal using a Gaussian blur filter with a radius of 2 pixels and a triangle threshold to convert the image to binary and create a mask. The resulting mean intensity in the define region of interest (ROI) for each condition was analysed with the GraphPad Prism 10.3 software. Statistical differences between groups were determined by the unpaired nonparametric Mann-Whitney test.

**RNA interference**

RNAi bacterial clones were obtained from the Ahringer library (Kamath et al., 2003) and verified by sequencing. RNAi bacteria were seeded on NGM plates supplemented with 100 g/ml ampicillin and 1 mM Isopropyl-β-D-thiogalactopyranoside (IPTG). Worms were transferred onto RNAi plates as L1 larvae and cultured at 25 °C until the young adult stage. In all our experiments, we used *sta-1* as a control, as we have shown that it does not affect the development nor any stress or innate response in the epidermis (Aggad et al., 2023).

**Wounding**

Needle wounding was performed as previously described (Pujol et al., 2008a; Taffoni et al., 2000) with a standard microinjection needle under a dissecting microscope by pricking the worm's posterior body or tail on agar plates; worms were analysed after 4 to 8 h.

## Reagents

PCR and sequencing primers used for *syb1965* where:

HB20-seq-s：TGTTCTTGTCGTCCTTCTCG

HB20-seq-a：CCATGTCTCAGTTGCCTTA

PCR and sequencing primers used for *syb1939* where:

HB19-seq-s: CGTCATCATTTGGTTCGTCA

HB19-seq-a: TCCTTTGGTCTCGAAATGAC

HB19-mid-s: CTGGGAGCGTGTCACCACCTA
